# Construction and multicohort validation of a colon cancer prognostic risk score system based on big data of neutrophil-associated differentially expressed genes

**DOI:** 10.7150/jca.94560

**Published:** 2024-03-25

**Authors:** Yunxi Yang, Cheng Lu, Linbin Li, Chunfang Zheng, Yifan Wang, Jiahui Chen, Bingwei Sun

**Affiliations:** Research Center for Neutrophil Engineering Technology, The Affiliated Suzhou Hospital of Nanjing Medical University, Suzhou 215002, Jiangsu Province, China.

**Keywords:** neutrophil, colon cancer, molecular subtypes, prognosis risk score, immunotherapy

## Abstract

**Objective:** To investigate the role of neutrophils in colon cancer progression.

**Methods:** Genetic data from 1,273 patients with colon cancer were procured from public databases and categorized based on genes linked to neutrophils through an unsupervised clustering approach. Through univariate Cox regression analysis, differentially expressed genes (DEGs) influencing overall survival (OS) were identified, forming the basis for establishing a prognostic risk score (PRS) system specific to colon cancer. Additionally, the correlation between PRS and patient prognosis, immune cell infiltration, and intratumoral gene mutations were analyzed. Validation of PRS as an indicator for "pan-tumor" immunotherapy was conducted using four distinct immunotherapy cohorts.

**Results:** The research identified two distinct subtypes of colon cancer, namely Cluster A and B, with patients in Cluster B demonstrating remarkably superior prognoses over those in Cluster A. A total of 17 genes affecting OS were screened based on 109 DEGs between the two cluster for constructing the PRS system. Notably, individuals classified under the high-PRS group (PRS_high_) exhibited poorer prognoses, significantly linked with immune cell infiltration, an immunosuppressive tumor microenvironment, and increased genomic mutations. Remarkably, analysis of immunotherapy cohorts indicated that patients with PRS_high_ exhibited enhanced clinical responses, a higher rate of progression-free events, and improved overall survival post-immunotherapy. The PRS system, developed based on tumor typing utilizing neutrophil-associated genes, exhibited a strong correlation with prognostic elements in colon cancer and emerged as a vital predictor of "pan-tumor" immunotherapy efficacy.

**Conclusions:** PRS serves as a prognostic model for patients with colon cancer and holds the potential to act as a "pan-tumor" universal marker for assessing immunotherapy efficacy across different tumor types. The study findings lay a foundation for novel antitumor strategies centered on neutrophil-focused approaches.

## Introduction

Cancer is the leading cause of premature death globally, posing a severe threat to human health 1. Colon cancer, ranking as the third most prevalent malignant tumor, affects millions of people worldwide, with its onset increasingly observed at a younger age 2, 3. Despite significant advances in the treatment and care of colon cancer 4, China has witnessed a sharp surge in its incidence and mortality rates in recent years 5. Hence, improving universal health coverage and establishing prognostic assessment indicators and targeted therapy for colon cancer are imperative to enhance the prognosis of patients with colon cancer.

**Figure 1:**
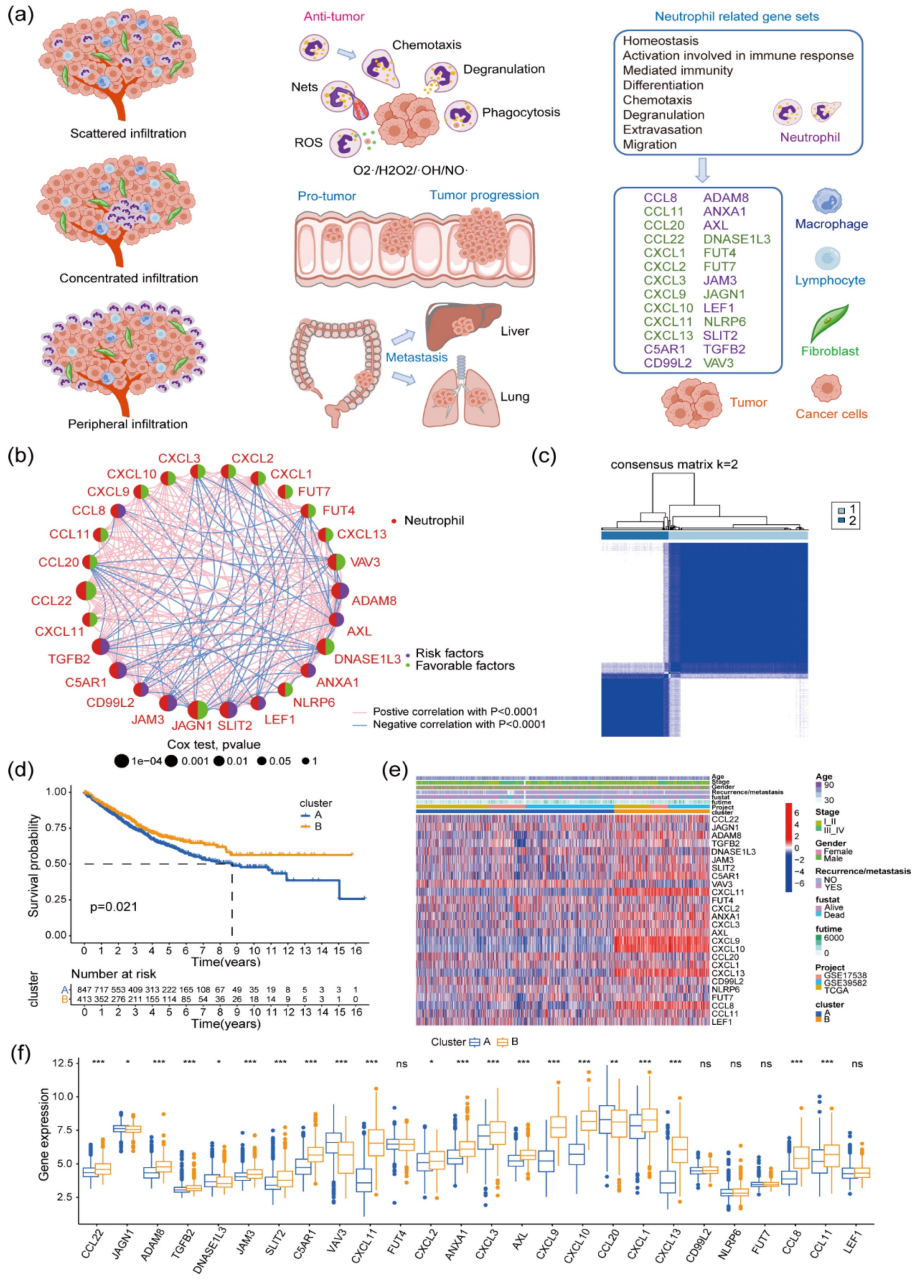
** Subgroup typing of patients with colon cancer based on neutrophil-associated genes. (a)** Distribution status of tumor-associated neutrophils within colon cancer, pro- and anti-cancer roles, and screening of prognostic genes of neutrophil origin within colon cancer; **(b)** Correlation between the expression levels of 26 prognostic neutrophil-specific genes in the tumor tissues of patients with colon cancer (N = 1273). Circle size represents the *P*-value of the Pearson's test for prognostic correlation between the relevant genes and patients with colon cancer; the right half of the circle suggests patient survival-related characteristics (purple denotes prognostic risk factors; green denotes prognostic protective factors); the line between the circles represents the correlation between the expression of the two genes (red represents a positive correlation with *P* < 0.0001, and blue represents a negative correlation with *P* < 0.001);** (c)** Unsupervised cluster analysis of patients with colon cancer (N = 1273) based on the expression of 26 genes, yielding two subtypes (Clusters A and B); **(d)** Prognostic differences in patients with colon cancer with different subtypes (log-rank test, *P* = 0.021); (E) Heatmap of gene expression levels and related clinical features between the colon cancer clusters; (F) Differential gene expression of 26 tumor-associated neutrophils between the different subtypes of colon cancer; ns, no significance: *P* > 0.05, **P < 0.01,***P < 0.001.

Neutrophils, also recognized as polymorphonuclear leukocytes (PMNs), are widely present in the peripheral circulation, serving as the most abundant innate immune cells and acting as first-line responders in the immune system, defending the host against invading pathogens 6, 7. These neutrophils can infiltrate tumor tissues, known as tumor-associated neutrophils (TANs). TANs demonstrate a dual role in promoting and suppressing tumors during tumorigenesis, progression, metastasis, and potential recurrence because of their adaptable phenotype and functionality 8-10. Elevated TANs in colon cancer correlate with a poorer patient prognosis 11. According to TAN-related literature 12-15, the infiltration pattern of TANs in colon cancer is categorized into three types: scattered infiltration, concentrated infiltration, and peripheral infiltration. TANs exhibit their antitumor effects by phagocytosis, releasing reactive oxygen species, degranulation with proteases (e.g., myeloperoxidase, elastase), and generating neutrophil extracellular traps (NETs) 8, 16, 17. Additionally, they can stimulate colon cancer progression and liver and lung metastasis by promoting the epithelial-mesenchymal transformation of tumor cells 18, angiogenesis 19, 20, enhancing inflammation 21, trapping circulating tumor cells through NETs 22, and increasing vascular permeability 23 (Figure 1a). Neutrophil heterogeneity causes temporal and spatial variability, contributing to their multifaceted and contradictory roles in colon cancer. Therefore, colon cancer subtypes studies based on neutrophil-associated genes could aid in targeted evaluation and personalized treatment for this disease.

In this study, 183 neutrophil-associated genes were gathered from a public data platform. Subsequently, 26 genes significantly associated with OS and prognosis of patients with colon cancer were identified. Unsupervised cluster analysis was conducted on 1,273 patients with colon cancer based on these genes. Additionally, 17 genes affecting overall survival were summarized from DEGs in patients with colon cancer with different subtypes to establish the PRS system. The investigation delved into the relationship between PRS and patient prognosis, immune cell infiltration, intratumoral gene mutations, and the efficacy of immunotherapy. Finally, the performance of PRS as a "pan-tumor" model of immunotherapy efficacy was validated across four independent immunotherapy cohorts. Molecular typing and PRS unveiled the characteristics of the immune microenvironment of patients with colon cancer. Overall, these findings offer novel insights into the prognostic assessment and clinical management of patients with colon cancer.

## Methods

### Data sources and processing of patients with colon cancer

The effect of neutrophil-specific genes on the prognosis and survival of patients with colon cancer was assessed by analyzing data from three clinical prognosis-related patient cohorts: The Cancer Genome Atlas (TCGA)-Colon Adenocarcinoma (COAD) dataset (469 patients), sourced from the UCSC-XENA database (https://xenabrowser.net/datapages/), GSE17538 (238 patients), and GSE39582 (566 patients; https://www.ncbi.nlm.nih.gov/geo/). Clinical data were retrieved from the corresponding Gene Expression Omnibus databases. Gene expression underwent batch correction using the Combat algorithm 24, yielding 16,928 gene expression data points for 1273 patients with colon cancer. The detailed clinic parameters of the enrolled patients were listed in Supplementary Table 1.

### Neutrophil-associated genes and colon cancer typing

Neutrophil-specific expressed genes were obtained by querying the MsigDB database for Gene Ontology (GO) annotation-related gene sets. The GO biological process (GO-BP) database was searched with neutrophil as the keyword. Eight significant gene sets associated with neutrophil function were identified, including GOBP NEUTROPHIL HOMEOSTASIS, GOBP NEUTROPHIL ACTIVATION INVOLVED IN IMMUNE RESPONSE, GOBP NEUTROPHIL MEDIATED IMMUNITY, GOBP NEUTROPHIL DIFFERENTIATION, GOBP NEUTROPHIL CHEMOTAXIS, GOBP NEUTROPHIL DEGRANULATION, GOBP NEUTROPHIL EXTRAVASATION, and GOBP NEUTROPHIL MIGRATION. Gene integration for each gene set revealed that the eight gene sets incorporated a total of 183 independent genes. The relationship between these genes and the survival and prognosis of patients with colon cancer was examined based on optimal cutoff values of the related genes using the surv_cutpoint function of the survminer package 25. Patients were stratified into high- and low-expression groups. based on relevant cutoff values. Univariate Cox regression analysis was conducted to determine the hazard ratio of gene expression levels on the overall survival and prognosis of patients with colon cancer. To minimize false-positive results, a threshold of *P* < 0.01 was set for significant prognostic genes. Among these genes, 26 were notably linked to the survival and prognosis of patients with colon cancer. Based on the expression levels of these 26 genes, a subgroup analysis of patients with colon cancer was performed by applying unsupervised clustering using ConsensusClusterPlus in R Language 26, grouping patients into Clusters A and B. A list of the neutrophil-associated genes within each GO-BP gene set is presented in Supplementary Table 2.

### Analysis of DEGs between colon cancer subtypes

DEGs between Clusters A and B were identified using the "limma" package in R, applying criteria of an adjusted *P* value < 0.001 and |logFC| > 1. Further analysis using the survminer package revealed 17 genes significantly associated with the survival and prognosis of patients with colon cancer. These 17 genes led to the categorization of colon cancer into two subgroups, named GeneClusters A and B, respectively, through ConsensusClusterPlus in R Language.

### PRS construction

Principal component analysis (PCA) was conducted utilizing the “prcomp” function in the R package “stats” for dimensionality reduction. The outcomes of PCA were depicted using the “ggbiplot” of the R package. The combined value of PC1 and PC2 was designated as the PRS. The cohort was divided into two groups, PRS_high_ and PRS_low_, based on the median risk score. Subsequently, the disparity in survival between the two patient groups was compared using the Kaplan-Meier plot and log-rank test.

### Gene set enrichment and correlation analyses

Gene-set variation analysis (GSVA) enrichment was carried out to explore the diversity in various biological processes employing the “GSVA” package. A heatmap illustrating the intensity of gene set enrichment was generated using the pheatmap package. Following this, the level of immune cell infiltration in tumor tissues was evaluated utilizing a compilation of 23 commonly expressed gene sets specific to immune cells, as summarized by Bindea et al 27. Furthermore, the correlation between relevant immune cell infiltration levels and patient PRS was analyzed.

### Mutation analysis of genomic data from TCGA-COAD patients

The mutation annotation format from the TCGA database was generated utilizing the R package “maftools,” and the mutations in PRS_high_ and PRS_low_ were plotted. Additionally, the tumor mutational burden (TMB) for each patient with COAD in the TCGA cohort was calculated. The mRNA expression-based stemness index (mRNAsi) and DNA methylation-based stemness index (mDNAsi) were employed to reflect the gene expression and epigenetic characteristics of stem cells, respectively, and measure the correlation between these characteristics and PRS, according to the one-class logistic regression (OCLR) machine learning algorithm reported by Malta et al 28.

### Clinical immunotherapy population cohort

Four immunotherapeutic cohorts were employed to validate the prediction of immunotherapy efficacy based on PRS: (1) patients with melanoma receiving anti-PD-1 (GSE78220, 26 cases); (2) non-small cell lung cancer patients receiving anti-PD-1/PD-L1 therapy (GSE135222, 27 cases); (3) bladder cancer patients receiving Bacillus Calmette Guerin (BCG) vaccine immunotherapy (GSE176307, 90 cases); and (4) patients with upper urinary tract tumor receiving anti-PD-L1 immunotherapy (IMvigor210CoreBiologies, 348 cases; http://research-pub.gene.com/IMvigor210CoreBiologies/). The relationship between patient stratification, survival, and tumor prognosis under PRS optimal cutoff conditions was analyzed using survminer software. Additionally, the difference in survival between the two groups was compared using the Kaplan-Meier plot and log-rank test.

### Chemotherapy drug sensitivity analysis

Colon cancer sensitivity to 138 chemotherapeutic drugs was evaluated using the pRRophetic algorithm software R package developed by Geeleher et al 29. alongside gene expression profiles of patients with colon cancer. Moreover, the significance of the difference in IC_50_ of drug sensitivity between the PRS_high_ and PRS_low_ groups was compared using the nonparametric Wilcox-test.

### Statistical analyses

All correlation analyses in this study were statistically assessed using R software (version 4.2.2; www.r-project.org) and associated software packages. Pearson's correlation coefficient was used for correlation analysis between variables. Differences in gene expression between cell subpopulations, PRS, and immune infiltration levels were examined using the Wilcoxon test. Between-group disparities in categorical variables were evaluated using the chi-square test. Unless otherwise specified, all tests were two-sided, and *P* < 0.05 denoted significant differences.

## Results

### Value of neutrophil-specific genes in colon cancer subgroups

The prognostic value of 183 neutrophil-associated genes (Supplementary Table 2) was initially assessed in 1,273 patients with colon cancer (Supplementary Table 3). Out of these, 26 genes were significantly associated with patient prognosis (univariate Cox regression *P* < 0.01; Figure 1a, Supplementary Figure 1). Among them, 10 genes were identified as unfavorable prognostic factors (ADAM8, ANXA1, AXL, C5AR1, CCL8, CD99L2, JAM3, LEF1, SLIT2, and TGFB2). Notably, a significant positive correlation was observed in the expression levels among these genes. On the contrary, the remaining 16 genes (CCL11, CCL20, CCL22, CXCL1, CXCL2, CXCL3, CXCL9, CXCL10, CXCL11, CXCL13, DNASE1L3, FUT4, FUT7, JAGN1, SLIT2, and TGFB2) were favorable prognostic factors, showing a significant positive correlation in their expression levels (*P* < 0.05). Furthermore, a significant negative correlation was observed between the expression levels of the 16 favorable genes and the 10 unfavorable genes. Subsequent unsupervised cluster analysis of patients with colon cancer based on the expression levels of these 26 genes resulted in two major Clusters (Cluster A and B; Figure 1B). The survival and prognostic analyses of patients indicated a significant difference in overall survival between the two groups, with patients in Cluster B having a significantly better prognosis than those in Cluster A (log-rank *P* = 0.021; Figure 1c). Further investigation revealed differences in neutrophil gene expression between the two clusters. Genes such as JAGN1, DNASE1L3, VAV3, and CCL20 were expressed at lower levels in Cluster B, while 17 genes including CCL12, ADAM8, TGFB2, and JAM3 were expressed at relatively higher levels in Cluster B (Figure 1d-e). Additionally, Clusters A and B displayed differences in the enrichment degree of multiple gene sets related to various signaling pathways, as observed in the HALLMARK, KEGG, and Reactome gene sets (Supplementary Figure 2). In the HALLMARK gene set, Cluster B exhibited higher enrichment in several signaling pathways, including Interferon-γ, Interferon-α, IL6-JAK-STAT3, Angiogenesis, and Hypoxia. Conversely, peroxisome and Wnt/β-catenin signaling pathways displayed greater enrichment in Cluster A. Within the KEGG gene set, Cluster B demonstrated increased enrichment in NOD-like-receptor, T-cell receptor (TCR), natural killer cell-mediated cell killing, and cell adhesion signaling pathways. In the reactome gene set, Cluster B exhibited higher enrichment in multiple signaling pathways, such as interleukin (IL)-12, TCR, IL-10, and PD-1.

### Cluster immune infiltration and differential gene expression analyses in patients with colon cancer

**Figure 2:**
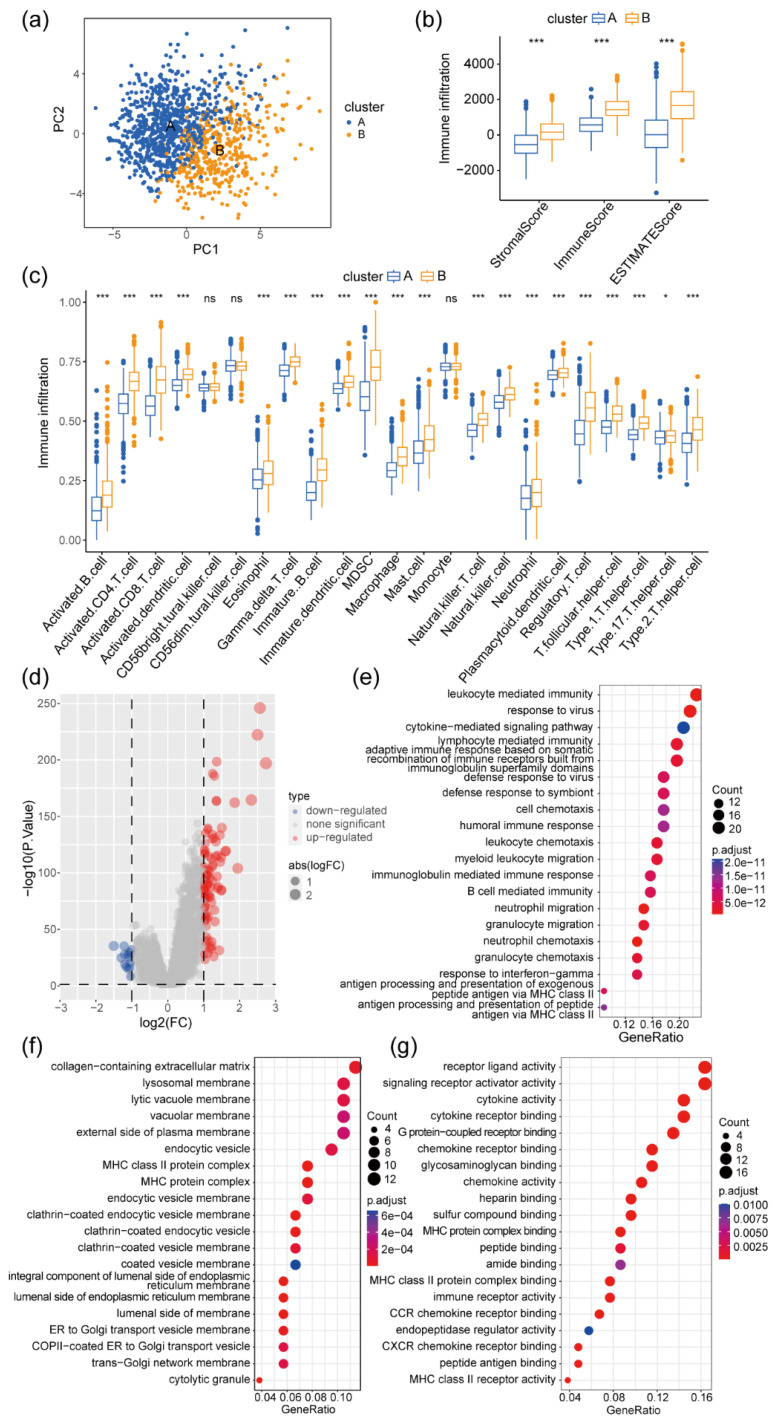
** Differences in immune infiltration and gene expression levels between the two clusters of patients with colon cancer. (a)** PCA of the two clusters.** (b)** Differences in cellular stroma, immune infiltration, and tumor purity values between the two clusters of patients with colon cancer, ***P < 0.001; **(c)** Differences in immune cell infiltration scores between the two clusters of patients with colon cancer, ns, no significance: *P* >0.05, *P <0.05, **P <0.01, ***P <0.001. **(d)** Volcano plot of the differentially expressed genes (DEGs) between the two clusters of patients with colon cancer. Red represents genes with high expression in Cluster B, blue represents genes with low expression in Cluster B, and DEGs with |logFC|>1 and P-false discovery rate (FDR) < 0.05 are considered significant DEGs; **(e-g)** Functional enrichment (Gene Ontology) analysis of 109 DEGs between two clusters of patients with colon cancer (Clusters A and B), covering biological process (BP) **(e)**, cellular component (CC) **(f)**, and molecular function (MF) **(g)**. The analysis results were sorted according to the GeneRatio size of the pathway, where the circle size represents the number of genes enriched, and the color represents the *P*-value of the enrichment analysis.

The distribution of the two clusters in PCA is shown in Figure 2a. Numerous studies have underscored the significant correlation between the infiltration of immune and stromal cells within tumor tissue and the survival rates and prognosis of patients with tumor 30-32. Utilizing the ESTIMATE algorithm, the StomalScore, ImmuneScore, and ESTIMATEScore of patients in Clusters A and B were compared. Cluster B exhibited significantly increased levels of stromal and immune cell infiltration, as well as total infiltration of both cell types, in contrast to Cluster A (Figure 2b). Moreover, employing GSVA, the infiltration levels of 23 immune cell types in colon cancer tissues were assessed for both clusters. Cluster B exhibited significantly higher infiltration levels in 20 out of 23 immune cell subpopulations, including B cells, T cells, monocytes, and macrophages, compared with Cluster A (Figure 2c). Therefore, patients with elevated levels of immune and stromal cell infiltration in colon cancer tissues experienced significantly improved survival and prognosis. By examining gene expression differences between Cluster A and B, 94 significantly highly expressed genes and 15 significantly low expressed genes were identified in Cluster B relative to Cluster A (Figure 2d). Biological process (BP) GO enrichment analysis highlighted the involvement of these DEGs primarily in immune responses mediated by lymphocytes, B cells, neutrophils, and myeloid cells (Figure 2e). Cellular component (CC) GO enrichment analysis revealed significant enrichment of DEGs in signaling pathways such as extracellular matrix collagen, endocytosis vesicle, and Golgi-related pathways (Figure 2f). Molecular Function (MF)-related signaling showed significant enrichment of related genes in pathways such as cytokine receptors, MHC-II protein molecules, CCR cytokine receptors, and CXCR receptors (Figure 2g).

### PRS construction

**Figure 3:**
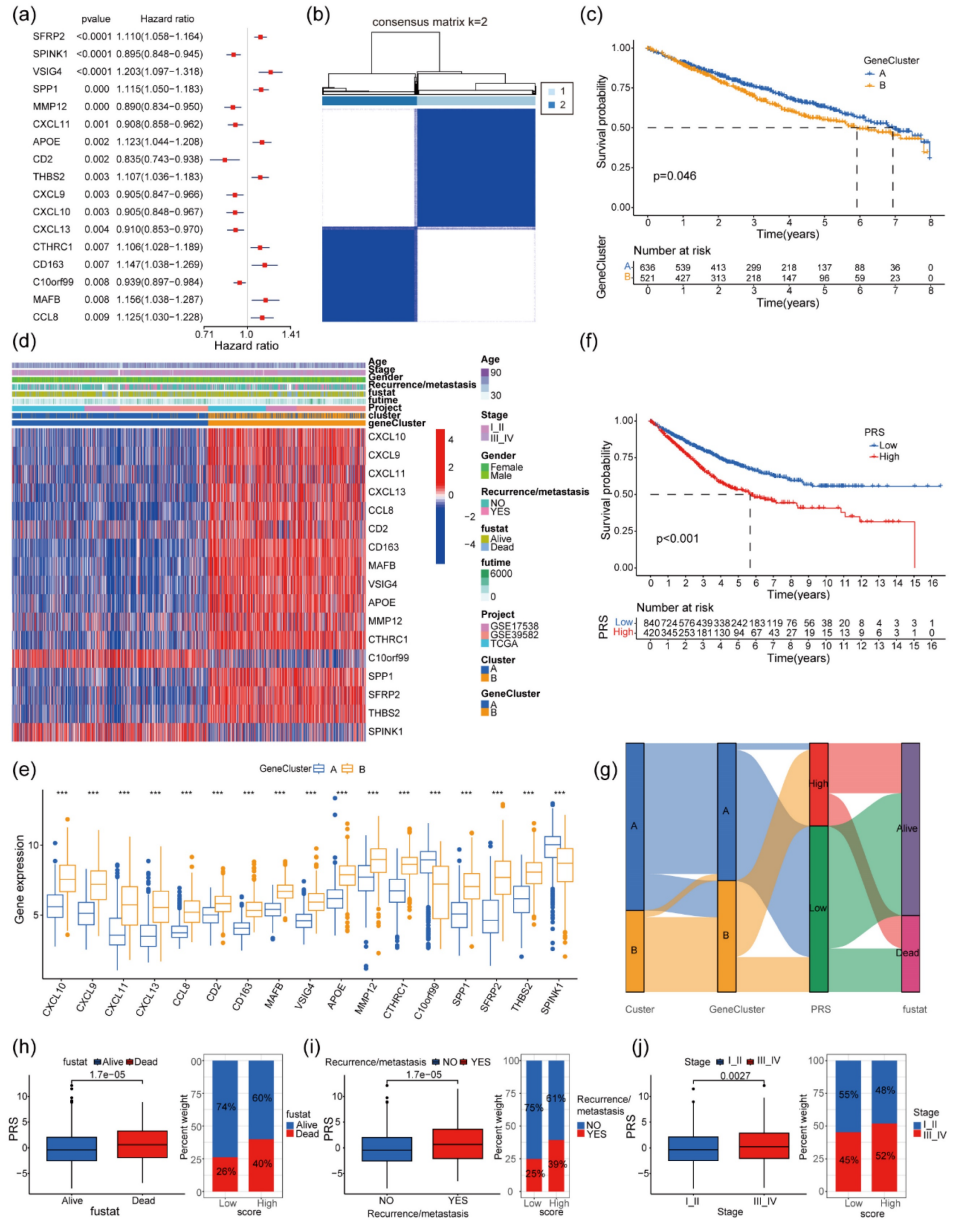
** Correlation analysis of DEGs between two GeneClusters of patients with colon cancer with their clinical characteristics. (a)** Forest plot showing 17 DEGs associated with survival in patients with colon cancer (univariate Cox regression *P* < 0.01); **(b)** Unsupervised cluster analysis of colon cancer based on 17 prognostic genes, categorizing patients with colon cancer into two gene clusters (GeneClusters A and B); **(c)** Kaplan-Meier plots showing the differences in survival among patients after clustering (log-rank test *P* = 0.046); **(d)** Heatmap showing gene expression levels and related clinical features between two gene clusters of patients with colon cancer; **(e)** Expression of 17 prognostic genes in the two gene clusters (*** *P* < 0.001); **(f)** Survival curves of patients with PRS_high_ and those with PRS_low_ (log-rank test *P* < 0.001); **(g)** Sankey diagram showing the distribution of prognostic status of Clusters A and B, GeneClusters A and B, and patients with PRS_high_ and those with PRS_low_; and differences in survival status **(h)**, recurrence or metastasis status **(i)**, and clinical stage** (j)** between patients with PRS_high_ and those with PRS_low_.

The correlation between the identified 109 significant DEGs and the survival rates as well as the prognosis of patients with colon cancer was thoroughly analyzed (Supplementary Table 4). Notably, the tissue expression levels of 17 genes, including SFRP2, SPINK1, CXCL9, CXCL10, CD163, and e.g., demonstrated a significant correlation with the OS of patients (Figure 3a).

Employing unsupervised cluster analysis based on the expression levels of these 17 prognostic genes, patients were classified into two distinct groups, labeled as GeneCluster A and B (Figure 3b). The survival analysis indicated that patients belonging to GeneCluster A exhibited a significantly higher overall survival rate than those in GeneCluster B (Figure 3c, log-rank test *P* = 0.046). Moreover, an in-depth investigation into the expression patterns of these 17 prognostic genes within patient tumor tissues was conducted (Figure 3d). Except for C10orf99 and SPINK1, which showed lower expression in GeneCluster B, the remaining 15 genes exhibited relatively higher expression levels in GeneCluster B (Figure 3e). Utilizing these 17 genes, a PRS system was devised for patients with colon cancer. Patients were stratified into PRS_high_ and PRS_low_ groups based on the median risk score. Notably, patients with PRS_high_ displayed significantly lower overall survival rates than those with PRS_low_ (log-rank test *P* < 0.001, Figure 3f). The Sankey diagram suggested that patients in the PRS_high_ group predominantly belonged to the neutrophil-specific gene subgroup Cluster B and the 17 prognostic gene group (GeneCluster B). These patients also exhibited a higher percentage of follow-up mortality events (Figure 3g). Clinically, the PRS was relatively higher in patients with colon cancer who experienced follow-up events such as death, recurrence, metastasis, and clinical grade III/IV (Figure 3h-j).

### Correlation between PRS and immune cell infiltration in tissues of patients with colon cancer

**Figure 4:**
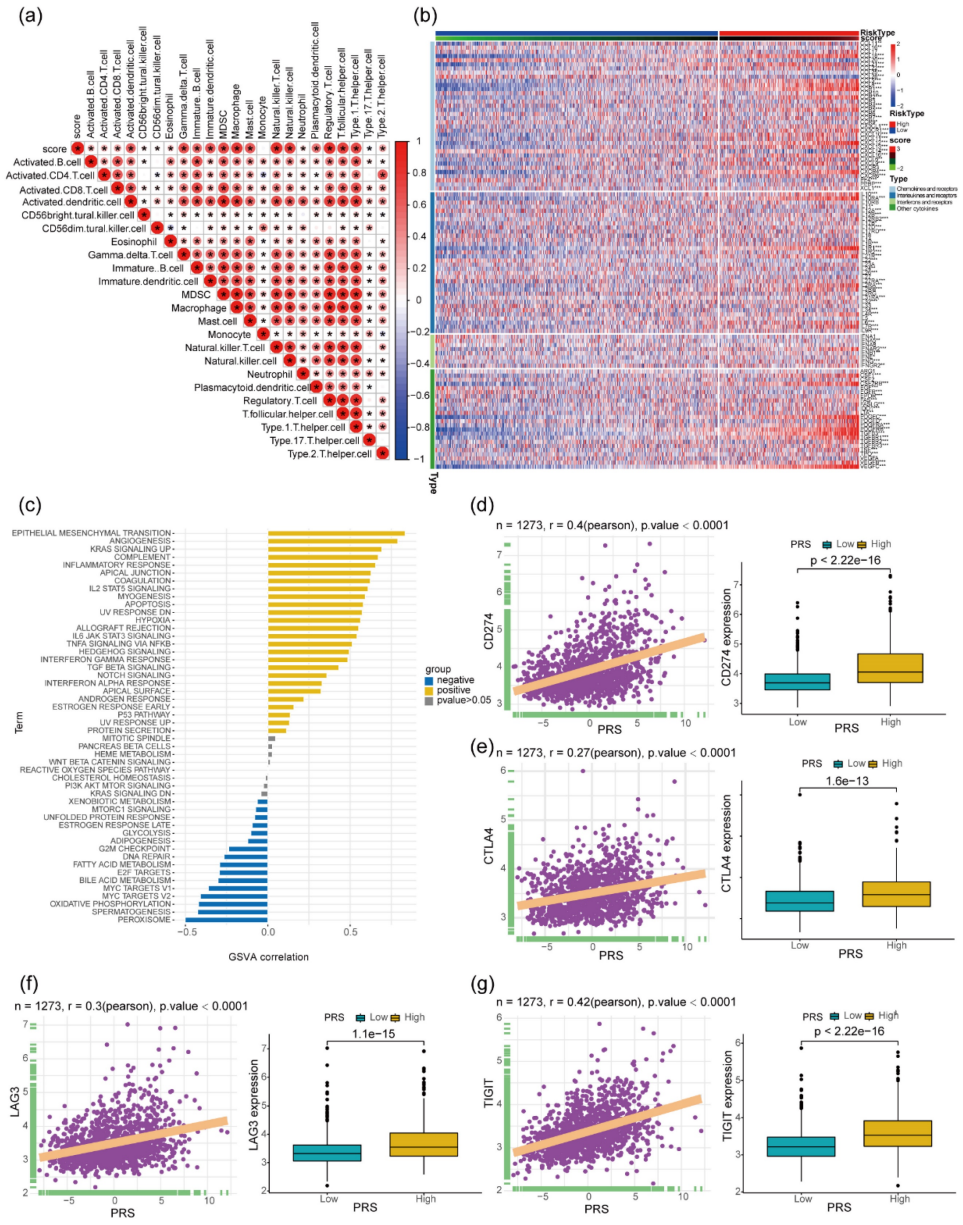
** Gene expression profiles of patients with PRS_high_ and PRS_low_ and their correlation with the expression of immunosuppressive molecules. (a)** Heatmap of the correlation between prognostic risk score (PRS) and immune cell infiltration in tumor tissues of patients with colon cancer. *P < 0.05; **(b)** Difference in the expression of chemokines and their receptors between patients with PRS_high_ and those with PRS_low_, ns > 0.05, *P < 0.05, **P < 0.01, ***P < 0.001; **(c)** Correlation of PRS with the enrichment level of 50 HALLMARK pathway genes. Significant positive correlation with *P* < 0.05 is denoted by yellow, and significant negative correlation with *P* < 0.05 is denoted by blue. **(d-g)** Significant positive correlation between PRS and expression of the immune checkpoint molecules CD274 **(d)**, CTLA4 **(e)**, LAG3 **(f)**, and TIGIT **(g)** (N = 1273); relatively higher expression of CD274, CTLA4, LAG3, and TIGIT in patients with colon cancer with PRS_high_ (Wilcoxon test *P* < 0.001).

Examining the infiltration levels of various immune cell types within colon cancer tissues revealed a significant positive correlation between PRS and the levels of immature B cells, immature dendritic cells (DC), myeloid-derived suppressor cells (MDSC), type 1-T_helper_, and type 17- T_helper_s (Figure 4a). Additionally, an analysis of the expression levels of chemokines, cytokines (ILs), interferons, and other immunomodulatory cytokines, and their receptors in colon cancer tissues demonstrated a notable positive correlation with PRS (Figure 4b). Subsequently, an assessment of HALLMARK gene set enrichment in colon cancer tissues revealed a significant positive correlation between PRS and pathways associated with tumor progression such as epithelial-to-mesenchymal-transition, Angiogenesis, and KRAS signaling pathways. Conversely, tumor suppressor-related signaling pathways such as peroxisome and oxidative phosphorylation showed a significant negative correlation with PRS (Figure 4c). In recent years, immunotherapeutic strategies targeting immune checkpoints have gained substantial attention. Notably, PRS exhibited a significant positive correlation with mRNA levels of immune checkpoint-associated molecules CD274, CTLA4, LAG3, and TIGIT in colon cancer tissues. Furthermore, the expression levels of related immunosuppressive molecules were significantly higher in patients with colon cancer and PRS_high_ (Figure 4d-g).

### Correlation between PRS and tumor genomic mutations in tissues of patients with colon cancer

**Figure 5:**
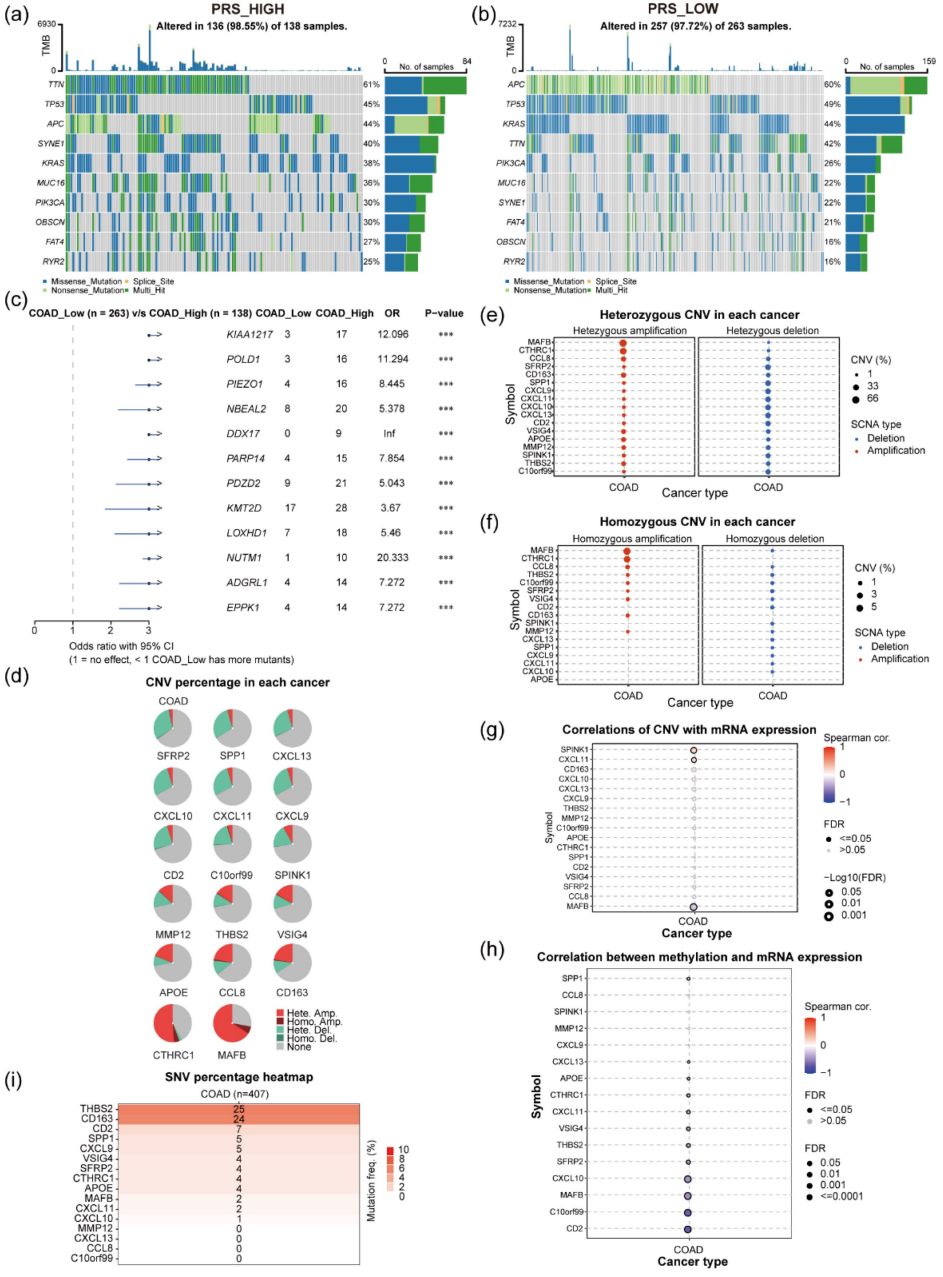
** Analysis of gene mutations, genomic variants, and regulation of methylation modifications in patients with colon cancer. (a)** Gene mutations in patients with colon cancer in the TCGA dataset PRS_high_ (N = 138); **(b)** Gene mutations in patients with colon cancer in the TCGA dataset PRS_low_ (N = 263); **(c)** Difference in gene mutation frequencies between patients with PRS_high_ and PRS_low_. **(d)** Percentage of gene amplifications and deletions of 17 genes in colon cancer tissues; **(e)** Percentage of heterozygous deletions or amplifications of 17 genes in colon cancer tissues; **(f)** Percentage of homozygous deletions or amplifications of 17 genes in colon cancer tissues; **(g)** Correlation analyses of copy levels of 17 genes and their mRNA expression levels in colon cancer tissues; **(h)** Correlation between genomic methylation levels and mRNA expression levels of 16 genes; **(i)** Mutation frequencies of 16 prognostic genes in patients with colon cancer. The circle size indicates the P value, and the color represents the correlation coefficient.

An investigation into the influence of gene mutations within tumor tissues on PRS involved a comparative analysis of gene mutation profiles between patients exhibiting PRS_high_ and PRS_low_ scores. Utilizing genomic mutation data from patients with colon cancer in the TCGA dataset, along with associated PRS (Figure 5a-b), revealed distinct mutation patterns. Among patients with PRS_high_ (N = 136), the most frequent gene mutations were observed in TTN (61%), TP53 (45%), and APC (44%; Figure 5a). By contrast, patients with PRS_low_ (N = 257) exhibited varying mutations, with the top 3 genes being APC (60%), TP53 (49%), and KRAS (44%; Figure 5b). Comparative analysis between these two clusters highlighted notable differences in gene mutations. Mutations in KIAA1217, POLD1, PIEZO1, NBEAL2, DDX17, and PARP14 were significantly more prevalent in patients with colon cancer and PRS_high_ than in those with PRS_low_ (Figure 5c). Moreover, examination of gene copy variants of 17 prognostic genes in colon cancer tumor tissues, utilizing the GSCA database (Figure 5d). MAFB and CTHRC1 showed significant gene amplification in colon cancer tissues (Figure 5e). However, SFRP2, SPINK1, CXCL11, CXCL13, and CXCL10 exhibited gene copy loss in certain tumor populations (Figure 5f). Further investigation into gene-mRNA correlations in colon cancer tissues unveiled intriguing relationships. Notably, SPINK1 and CXCL11 gene copy numbers exhibited a positive correlation with gene mRNA levels, while MAFB copy number displayed a negative correlation with its gene mRNA level in colon cancer tissues (Figure 5g). Additionally, methylation levels of specific genes such as CD2, C10orf99, MAFB, CXCL10, SFRP2, and THBS2 were found to have significant negative correlations with their corresponding gene expressions, while the methylation level of the SPP1 gene showed a positive correlation with its gene expression level (Figure 5h). The investigation delved into relevant gene variants in the TCGA-COAD dataset, revealing relatively low mutation frequencies for 17 prognostic genes in colon cancer. Only THBS2 and CD163 exhibited somatic mutations in 6.14% (25/407) and 5.90% (24/407) of patients with tumor, respectively, with the remaining genes showing mutation frequencies below 2% (Figure 5i).

Tumor cell stemness, a crucial factor in tumor progression and treatment outcomes 33, was assessed through mRNAsi and mDNAsi scores in TCGA-COAD colon cancer. Interestingly, both scores exhibited a significant negative correlation with PRS, with patients with PRS_high_ displaying lower mDNAsi and mRNAsi scores than those with PRS_low_ (Supplementary Figure 3a-b). Clinical parameters such as microsatellite instability (MSI) and TMB in colon cancer are closely associated with patients' clinical treatment options and prognosis 34, 35. A significant positive correlation was observed between PRS and patients' MSI scores in the TCGA-colon cancer population (N = 427; Pearson's r = 0.24, P < 0.001), with patients with PRS_high_ demonstrating higher MSI scores than those with PRS_low_ (Supplementary Figure 3C), and an increased tumor tissue TMB degree was observed in patients with colon cancer and PRS_high_ (Supplementary Figure 3D).

### Correlation of PRS with tumor clinical immunotherapy efficacy and chemosensitivity

**Figure 6:**
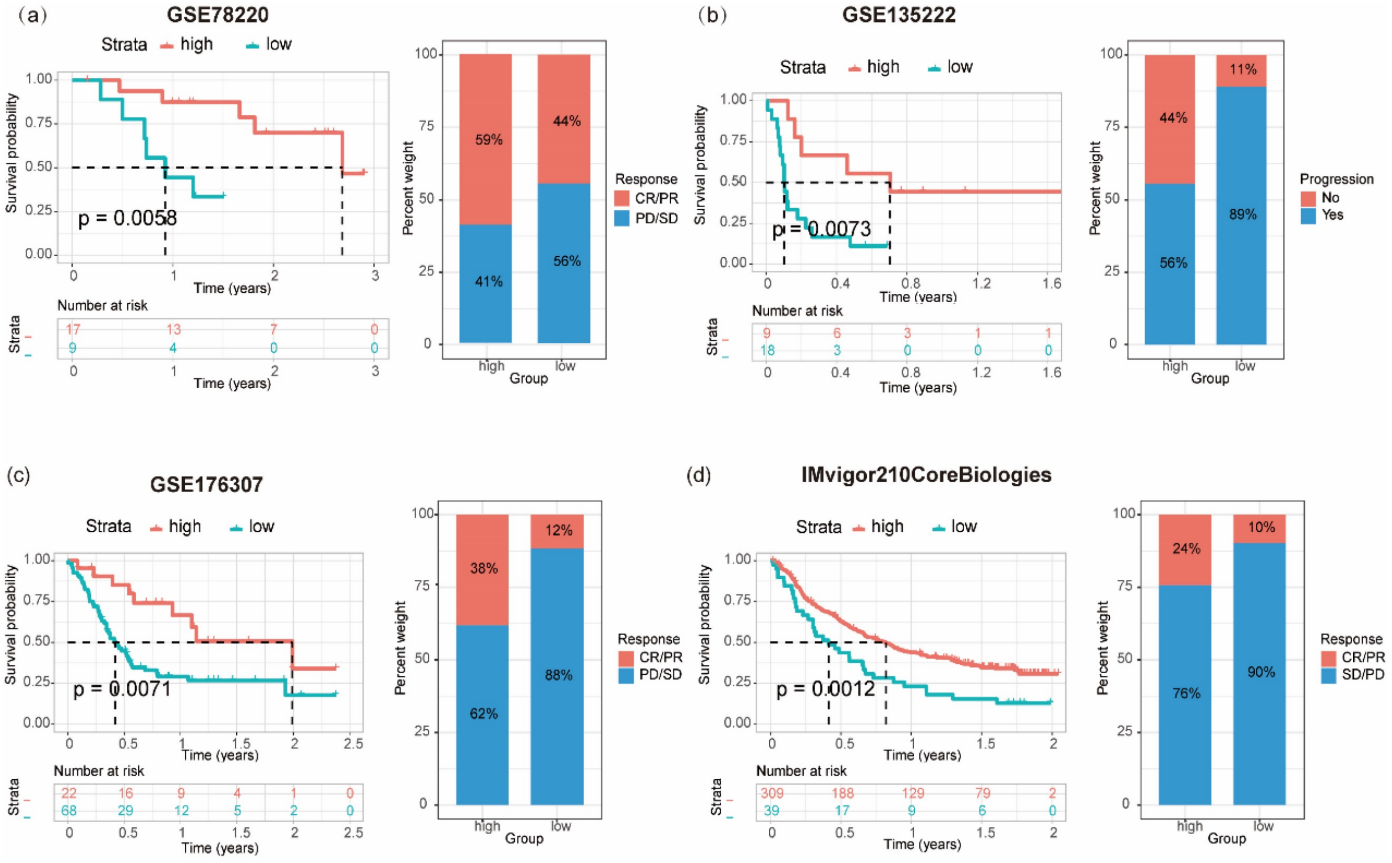
** Relationship between prognostic risk score (PRS) and the survival and prognosis of patients with tumor receiving immunotherapy.** Relationship between PRS and overall survival and immunotherapy efficacy (progressive disease/stable disease or partial response/complete response) in patients with melanoma **(a)**, bladder cancer** (c)**, and uroepithelial carcinoma **(d)**; **(b)** Association between PRS and disease-free survival and immunotherapy outcome (progression/non-progression) in patients with non-small cell lung cancer treated with anti-PD1/PD-L1.

Immunotherapy has emerged as a pivotal clinical treatment for tumors in recent years. A significant correlation was observed between the expression levels of patients' genes and the effectiveness of immunotherapy in a clinical setting. Seventeen genes derived from the colon cancer PRS were assessed as potential prognostic markers for "pan-tumor" immunotherapy within a publicly available dataset. A gene prognostic score, GPS, was constructed based on the expression patterns of these 17 genes. The predictive value of GPS regarding treatment outcomes and its association with patient clinical prognosis were subsequently evaluated across various cancer types. This evaluation encompassed patients receiving different forms of immunotherapy: patients with melanoma treated with anti-PD-1 (GSE78220; N = 26), patients with non-small cell lung cancer treated with anti-PD-1/PD-L1 therapy (GSE135222; N = 27), individuals with bladder cancer undergoing BCG immunotherapy (GSE176307; N = 90), and patients with upper urinary tract tumors treated with anti-PD-L1 immunotherapy (IMvigor210CoreBiologies; N = 348). In terms of clinical prognosis, individuals with PRS_high_ in melanoma (log-rank *P* = 0.0058; Figure 6a), bladder cancer (log-rank *P* = 0.0071; Figure 6c), and upper urinary tract epithelial tumors (log-rank *P* = 0.0012; Figure 6d) demonstrated a notably increased CR/PR (complete response/ partial response) and a significant decreased PD/SD (progressive disease/ stable disease) than those with PRS_low_. Within the non-small cell lung cancer patient cohort receiving immune checkpoint inhibitors, a relatively high proportion of patients with PRS_high_ experienced progression-free status. What's more, these patients also exhibited a significantly improved survival rate compared with those with PRS_low_ (log-rank *P* = 0.0073; Figure 6b).

To examine the effect of PRS on chemotherapy sensitivity in patients with colon cancer, the sensitivity of patients with tumor to 138 chemotherapeutic agents was assessed using the pRRophetic algorithm and colon cancer gene transcriptome data. Comparison between patients with PRS_high_ and PRS_low_ highlighted varying sensitivities to different compounds, with patients with PRS_high_ showing increased sensitivity to specific agents such as A-770041, ABT-263, AICAR, and AMG-706, while being less responsive to others such as A443654, AKT inhibitor, BIBW2992, and BIRB0796 (Supplementary Figure 4).

## Discussion

Colon cancer is one of the leading causes of cancer-related fatalities on a global scale, imposing substantial financial strains on national healthcare systems 23, 36. Tumors originate from chronic inflammation, with the tumor microenvironment characterized by persistent inflammation 37-39. Neutrophils, positioned at the intersection of inflammation and cancer, play a pivotal role in colon cancer. While most studies categorize neutrophils as short-lived cells 40, 41, circulating for a maximum of approximately 5.4 days 42, those present in tissues can survive for weeks 43. The prolonged lifespan of TANs implies adaptability and unpredictability in their functions. Research demonstrates that TANs exhibit diverse immune subtypes across different tumor types 44-47, discerned by functional or molecular markers. This spatial and temporal heterogeneity enables varied functionality at different tumor stages and regions. Daniel Triner et al. suggested that neutrophils could impede colon tumor growth and progression by limiting bacterial populations and tumor-associated inflammatory responses 21. Conversely, Zhang et al. suggested that neutrophils might promote colitis-associated colon carcinogenesis 48. Moreover, Rayes et al. found evidence that NETs could facilitate targeted metastasis in colon cancer 49. Nonetheless, the precise role of neutrophils in colon cancer remains incompletely characterized.

Neutrophil heterogeneity in colon cancer was analyzed in this study. Initially, patients with colon cancer were classified based on neutrophil-associated genes. Both patient clusters exhibited significant differences in gene expression levels and enrichment of associated signaling pathways. Remarkably, Cluster B patients exhibited superior prognosis and greater immune cell infiltration than Cluster A patients. Subsequently, 17 genes, significantly correlating with overall survival, were identified among 109 DEGs between the two clusters. A secondary unsupervised cluster analysis using these genes categorized patients into GeneCluster A and B. Patients in GeneCluster B demonstrated worse overall survival outcomes than those in GeneCluster A. The PRS system based on these 17 genes was developed to evaluate the prognosis of patients with colon cancer. Notably, patients with PRS_high_ scores exhibited significantly lower overall survival rates than those with PRS_low_ scores. Additionally, the PRS_high_ group experienced a higher rate of mortality, recurrence, metastasis, and clinical grade III/IV.

Further analysis focused on immune-related information. The PRS exhibited a significant positive correlation with the infiltration of diverse immune cells, expression levels of related cytokines, and mRNA levels of immune checkpoints CD274, CTLA4, LAG3, and TIGIT in colon cancer tissues. This suggests that infiltration by relevant immune cells could contribute to the poor survival prognosis in colon cancer patients with PRS_high_. The gene mutation profiles differed significantly between patients with PRS_high_ and those with PRS_low_, highlighting the significant effects of gene mutations and genomic methylation modifications on the PRS in patients with tumor. The PRS_high_ score group displayed increased immune cell infiltration, heightened expression of immune checkpoints and chemokine-related genes, greater gene mutations, and elevated TMB and MSI. Consequently, this patient cohort exhibited enhanced efficacy in immunotherapy. This was substantiated using independent immunotherapy cohorts, where patients with PRS_high_ displayed an increased CR/PR, a decreased PD/SD, a higher rate of progression-free events, and significantly improved overall survival. Overall, the PRS system can serve as a prognostic tool for patients with colon cancer and as a "pan-tumor" model for predicting immunotherapy effectiveness across various tumors. However, this study has certain limitations. The PRS model's development relied on a public database and lacked validation from prospective clinical trials.

The interactions between neutrophils and other cells in the tumor immune microenvironment, such as lymphocytes, macrophages, and fibroblasts, are of significant importance. Neutrophils exhibit both pro- and anti- tumor functions by differentially regulating components of the tumor microenvironment and other immune cells 50. Of the 17 genes constructing PRS, VSIG4 and CD163 genes, which are significantly associated with poor prognosis, are specifically expressed in macrophages 51, 52 VSIG4 is acknowledged as a promising macrophage immune checkpoint 53, while CD163 is deemed a specific marker for senescent macrophages 54. Investigating the role of intercellular communication between neutrophils and macrophages in colon cancer progression is crucial. Neutrophil heterogeneity gives rise to numerous immune subpopulations. Therefore, understanding the generation, evolution, temporal dynamics, and spatial phenotypic variations of neutrophil populations within the tumor microenvironment during colon cancer progression is imperative. Identifying and tracking changes in surface markers during the spatiotemporal dynamics of neutrophils and inducing the transformation of TANs into specific anti-tumor populations according to different subtypes and stages of the tumor are crucial aspects for successful tumor immunotherapy.

## Conclusions

In summary, PRS demonstrated significant associations with immune infiltration, immune checkpoints, gene mutations, TMB, MSI, immunotherapy efficacy, and prognostic features. Hence, PRS could serve as a prognostic model for patients with colon cancer, guiding personalized treatment. Additionally, PRS holds the potential to act as a "pan-tumor" universal marker for assessing immunotherapy efficacy across different tumor types.

## Supplementary Material

Supplementary figures.

Supplementary tables.
