# Supplementary material for: Construction and multicohort validation of a colon cancer prognostic risk score system based on big data of neutrophil-associated differentially expressed genes
Source: J Cancer. 2024 Mar 25;15(9):2866–79. doi: 10.7150/jca.94560 (PMC10988322; doi:10.7150/jca.94560)
Supplement: Supplementary file 1 — Supplementary figures. [file jcav15p2866s1.pdf]

Supplementary materials for

Construction and multicohort validation of a colon cancer prognostic risk score system based on neutrophil-associated differentially expressed genes

by

Yunxi Yang, Cheng Lu, Linbin Li, Chunfang Zheng, Yifan Wang, Jiahui Chen, Bingwei Sun

[*] Corresponding author

Email: sunbinwe@hotmail.com

This file includes:

Figures S1 to S4

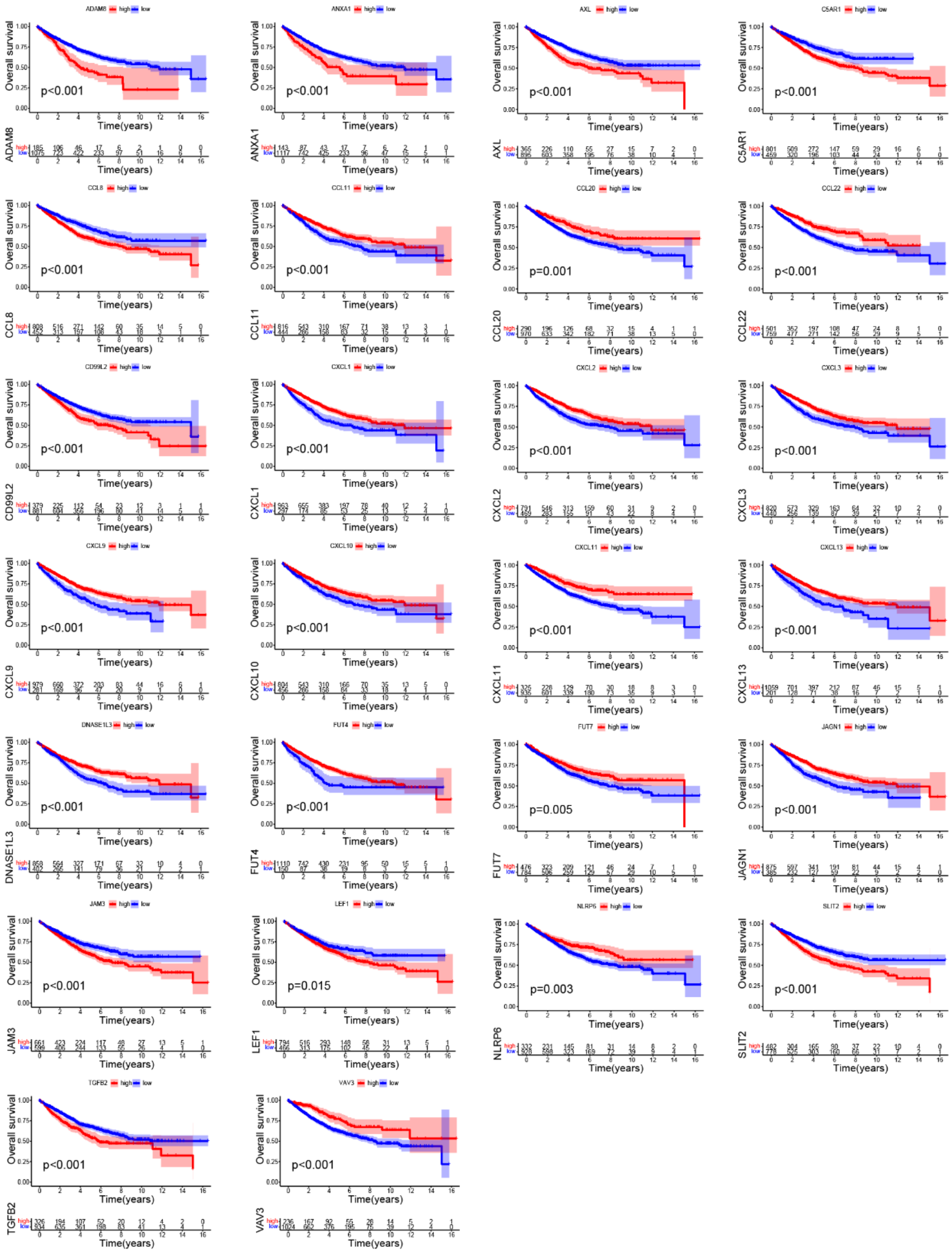

Supplementary figure 1: Neutrophil-associated genes and survival curves in patients with colon cancer.

Kaplan-Meier plot showing the correlation between 26 neutrophil-associated genes and survival in patients with colon cancer (log-rank test, $P < 0.001$).

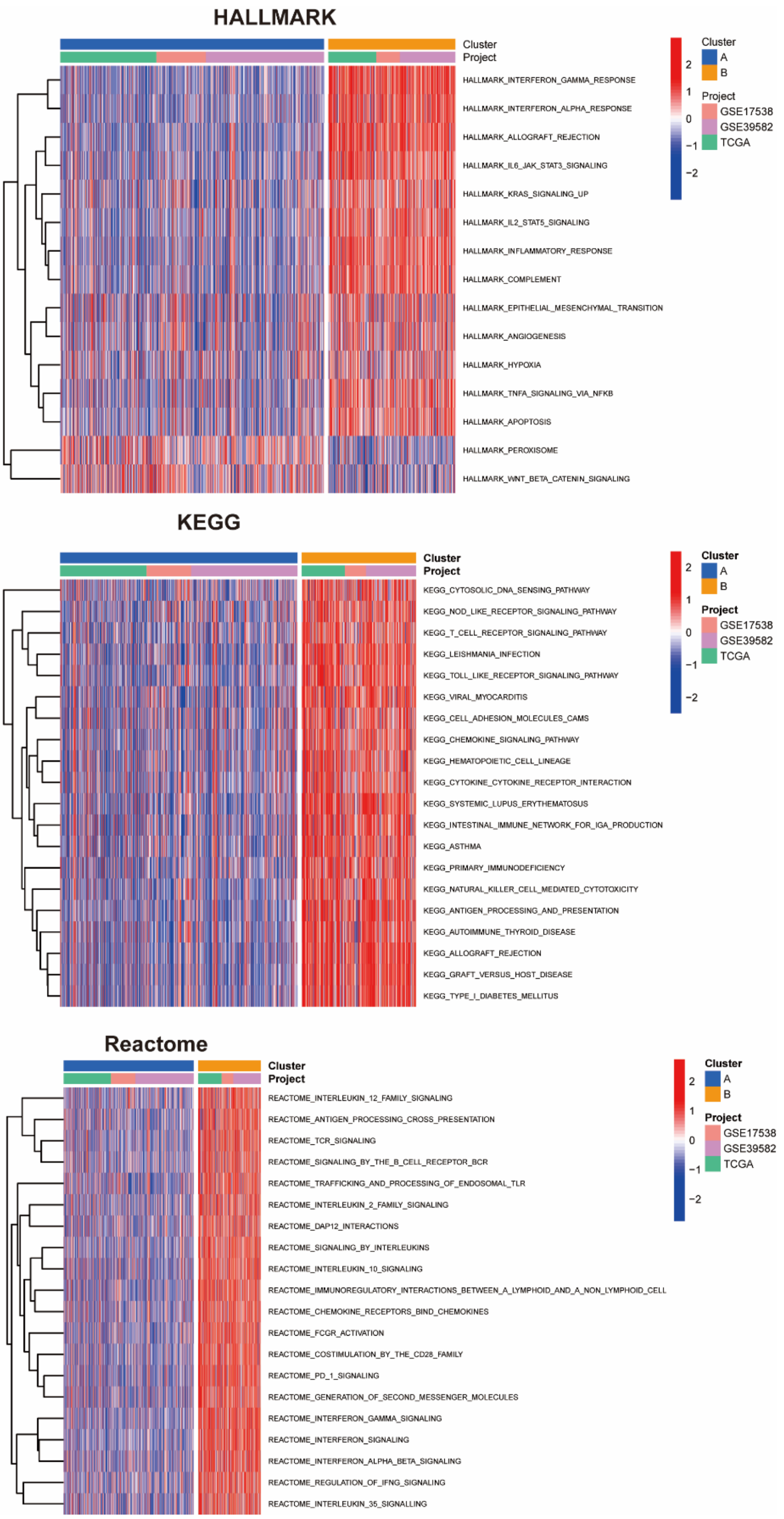

Supplementary figure 2: Analysis of differences in signaling pathway enrichment between the two clusters of patients with colon cancer.

Heatmap comparing the differential enrichment of HALLMARK, KEGG, and Reactome-related gene signaling pathways between Clusters A and B patients with colon cancer (FDR-P< 0.05 and |logFC| > 0.1).

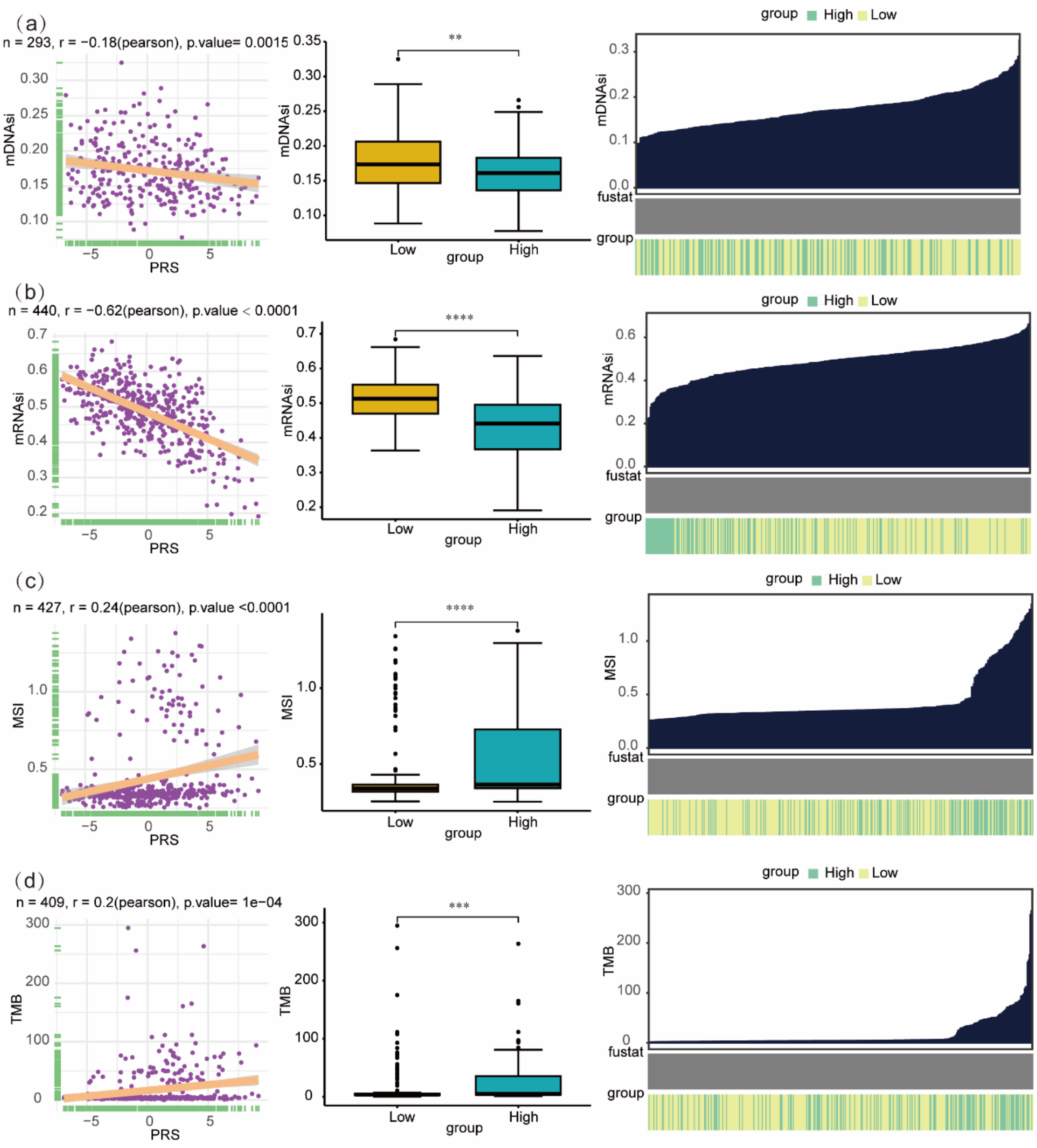

Supplementary figure 3: Correlation between PRS and tumor stemness, microsatellite instability, and tumor mutational burden in patients with colon cancer.

Correlation between PRS and mDNAsi score **(a)**, mDNAsi score **(b)**, MSI **(c)**, and TMB **(d)** (left); Comparison of mDNAsi score **(a)**, mDNAsi score **(b)**, MSI **(c)** and TMB **(d)** between patients with PRS_{high} and those with PRS_{low} (middle); Relationship between PRS and patient survival, mDNAsi score **(a)**, mDNAsi score **(b)**, MSI **(c)**, and TMB **(d)** (right).

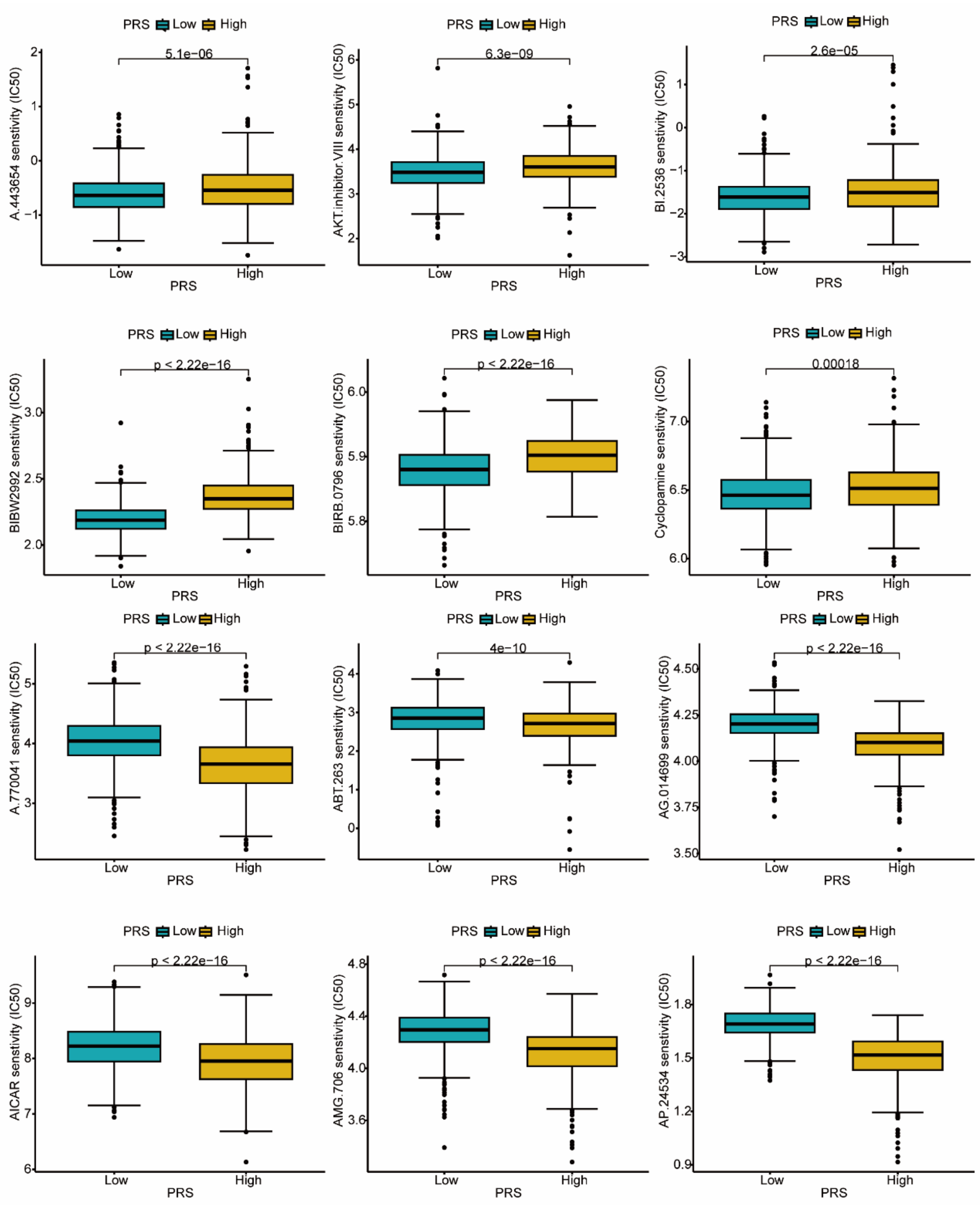

Supplementary Figure 4: Relationship between PRS and clinical chemotherapy sensitivity.

Differences in IC_{50} of 12 anticancer drugs between patients with PRS_{high} and those with PRS_{low}.
